# Characterization of poplar growth-regulating factors and analysis of their function in leaf size control

**DOI:** 10.1186/s12870-020-02699-4

**Published:** 2020-11-05

**Authors:** Jinnan Wang, Houjun Zhou, Yanqiu Zhao, Pengbo Sun, Fang Tang, Xueqin Song, Meng-Zhu Lu

**Affiliations:** 1 State Key Laboratory of Tree Genetics and Breeding, Key Laboratory of Tree Breeding and Cultivation of the National Forestry and Grassland Administration, Research Institute of Forestry, Chinese Academy of Forestry, Beijing, 100091 China; 2 Ludong University, Yantai, 264025 China; 3 Co-Innovation Center for Sustainable Forestry in Southern China, Nanjing Forestry University, Nanjing, 210037 China; 4 Zhejiang Agriculture & Forestry University, Hangzhou, 311300 China

**Keywords:** Growth-regulating factor, Phylogenetic relationship, miR396, Leaf development, *Populus*

## Abstract

**Background:**

Growth-regulating factors (GRFs) are plant-specific transcription factors that control organ size. Nineteen *GRF* genes were identified in the *Populus trichocarpa* genome and one was reported to control leaf size mainly by regulating cell expansion. In this study, we further characterize the roles of the other poplar GRFs in leaf size control in a similar manner.

**Results:**

The 19 poplar *GRF* genes were clustered into six groups according to their phylogenetic relationship with *Arabidopsis* GRFs. Bioinformatic analysis, degradome, and transient transcription assays showed that 18 poplar GRFs were regulated by miR396, with *GRF12b* the only exception. The functions of *PagGRF6b* (*Pag*, *Populus alba* × *P. glandulosa*), *PagGRF7a*, *PagGRF12a*, and *PagGRF12b*, representing three different groups, were investigated. The results show that *PagGRF6b* may have no function on leaf size control, while *PagGRF7a* functions as a negative regulator of leaf size by regulating cell expansion. By contrast, *PagGRF12a* and *PagGRF12b* may function as positive regulators of leaf size control by regulating both cell proliferation and expansion, primarily cell proliferation.

**Conclusions:**

The diversity of poplar GRFs in leaf size control may facilitate the specific, coordinated regulation of poplar leaf development through fine adjustment of cell proliferation and expansion.

**Supplementary information:**

**Supplementary information** accompanies this paper at 10.1186/s12870-020-02699-4.

## Background

Growth-regulating factors (GRFs) are plant-specific transcription factors that regulate the growth and development of leaves, roots, stems, flowers, and seeds by regulating cell proliferation or cell expansion, leading to the formation of larger organs [1–4]. GRFs form a multigene family found in the reported plant genomes: six genes in *Camellia sinensis*, eight genes in *Vitis vinifera*, nine genes in *Arabidopsis thaliana*, nine genes in *Citrus sinensis*, 10 genes in *Pyrus bretschneideri*, 12 genes in *Oryza sativa*, 13 genes in *Solanum lycopersicum*, 14 genes in *Zea mays*, 17 genes in *Brassica rapa*, 19 genes in *Populus trichocarpa*, and 25 genes in *Nicotiana tabacum* [5–14]. The Glu-Leu-Glu (QLQ) and Trp-Arg-Cys (WRC) domains are essential for GRF function in protein–protein interactions [15] and DNA binding [16], respectively. Genome-wide analyses revealed that GRFs and a few bZIP transcription factors are the major targets of miR396 [17].

GRFs are important for leaf size control [1–4]. Overexpression of *AtGRF1* (*At*, *Arabidopsis thaliana*), *rAtGRF2* (with mutations in the miR396 target sites, miR396-resistant version), *rAtGRF3*, *rAtGRF7*, *rAtGRF9*, *AtGRF5*, *BnGRF2* (*Bn*, *Brassica napus*), or *BrGRF8* (*Br*, *Brassica rapa*) in *Arabidopsis thaliana* and overexpression of *rZmGRF1* (*Zm*, *Zea mays*) in *Zea mays* all resulted in larger leaves, while *grf* mutations or overexpression of miR396 led to smaller leaves [6, 10, 18–22]. Interestingly, most reported GRFs (*AtGRF2*, *AtGRF3*, *AtGRF5*, *BnGRF2*, *BrGRF8*, and *ZmGRF1*) control leaf size by regulating cell proliferation [10, 15, 18, 20–22], except ZmGRF10, which modulates leaf size via both cell proliferation and cell expansion, but mainly through cell proliferation [23]. Previously, we found that overexpression of one of the poplar GRFs, GRF15, also led to larger leaves and further analysis revealed that this GRF controlled leaf size mainly by regulating cell expansion [24], which differed from the reported GRFs. Since 19 GRFs have been identified in the *Populus trichocarpa* genome [11] and leaf development is important for poplar biomass production in species like *P. nigra* and for drought/salt tolerance in species like *P*. *euphratica*, we wondered whether and how the other poplar GRFs function in leaf size control.

Here, we renamed the poplar GRFs according to their phylogenetic relationship with *Arabidopsis* GRFs and clustered them into six groups, and characterized the functions of *PagGRF6b*, *PagGRF7a*, *PagGRF12a*, and *PagGRF12b* from three different groups. We found that not all poplar GRFs regulate leaf development and their mechanisms of leaf size control are diverse.

## Results

### Names of poplar GRFs according to their *Arabidopsis* orthologs

**Fig. 1:**
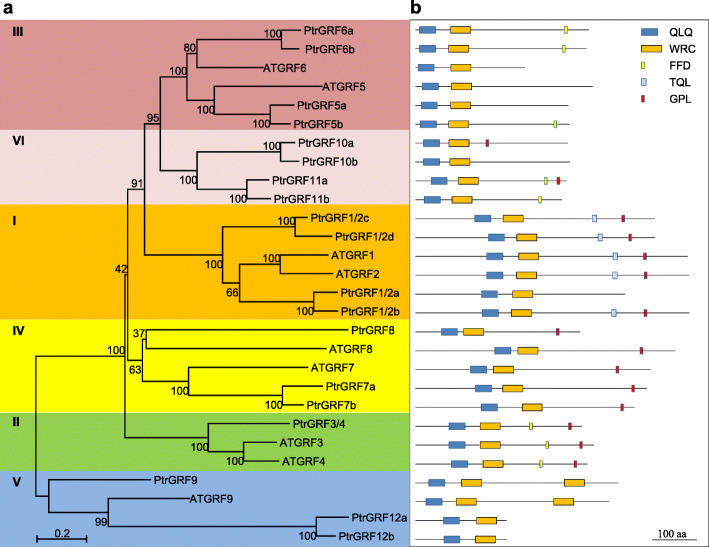
Phylogenetic relationships and gene structure of *A. thaliana* (At) and *P. trichocarpa* (Ptr) GRF genes. **a** The phylogenetic tree of AtGRFs and PtrGRFs. Full-length GRF protein sequences were aligned using Clustal X2.1 and a neighbor-joining phylogenetic tree was constructed using MEGA 5.0. The PtrGRFs were classified into six groups (marked with different background colors) according to the phylogenetic relationship. **b** Conserved domains or motifs in the GRF proteins. QLQ and WRC domains, FFD, TQL and GPL motifs (represented by boxes of different color) are named because of conserved Gln-Leu-Gln (QX_3_LX_2_Q), Trp-Arg-Cys (WRC), Phe-Phe-Asp (FFD), Thr-Gln-Leu (TQL) and Gly-Pro-Leu (GPL) residues contained in their sequences

Nineteen candidate *GRF* genes were found in the *Populus trichocarpa* genome [11]. To enable the comparison of *PtrGRFs* (*Ptr*, *Populus trichocarpa*) with the well-studied *AtGRFs*, the 19 identified *PtrGRFs* were renamed according to their *Arabidopsis* orthologs (Fig. 1, Fig. S1). According to the phylogenetic tree, the *PtrGRFs* could be classified into six groups (Fig. 1a, Fig. S2), with Group VI as a supplementary group to the reported classification of *AtGRFs* [6]. In Group I, four *PtrGRFs* clustered with *AtGRF1* and *AtGRF2* and were named *PtrGRF1/2a*, *PtrGRF1/2b*, *PtrGRF1/2c*, and *PtrGRF1/2d* (Fig. 1a). In Group II, only one *PtrGRF* gene corresponded to *AtGRF3* and *AtGRF4* and was named *PtrGRF3/4* (Fig. 1a). In Group III, *AtGRF5* and *AtGRF6* each have two poplar orthologs, which were named accordingly (Fig. 1a). In Group IV, three *PtrGRFs* were named according to their sequence similarity to *AtGRF7* and *AtGRF8* (Fig. 1a). In Group V, although three *PtrGRFs* clustered with *AtGRF9*, only one *PtrGRF* with two WRC domains was named *PtrGRF9* (Fig. 1a and b). In addition, four *PtrGRFs* with no close *Arabidopsis* orthologs were clustered in Group VI and named *PtrGRF10a*, *PtrGRF10b*, *PtrGRF11a*, and *PtrGRF11b* (Fig. 1a). The two *PtrGRFs* that clustered with *AtGRF9*, but lacked the additional WRC domain were renamed *PtrGRF12a* and *PtrGRF12b* (Fig. 1a and b). Table S1 shows the complete gene information for *PtrGRFs* and *AtGRFs*.

### The regulation of *PtrGRFs* by miR396

**Fig. 2:**
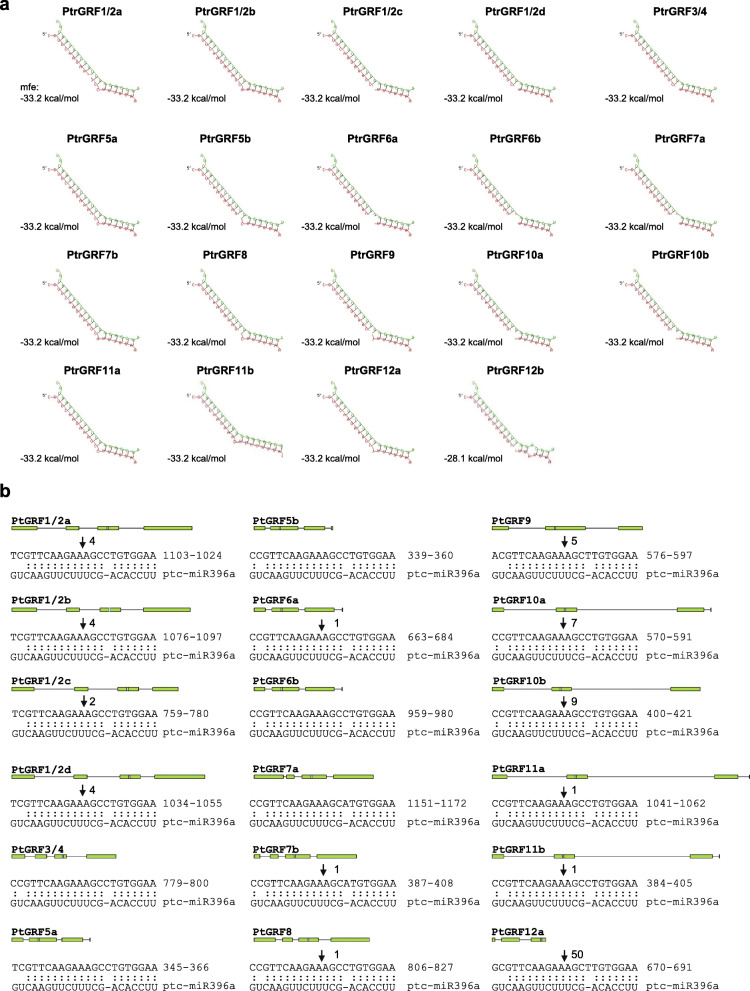
The relationship between miR396b and the PtrGRFs. **a** The hybridization of miR396b and PtrGRFs. The minimum free energy hybridization is shown. Characters in green and red are the nucleotide sequences of miR396 and GRFs, respectively. **b** Degradome data of the possible miR396b cleavage sites on PtrGRFs. The green boxed are the introns of GRFs. Bars indicate the location of the complementary nucleotides of GRFs to miR396. Arrows indicate the possible cleavage site. The raw reads detected by degradome analysis for each GRFs were marked next to the arrows

Since GRFs are the major targets of miR396 [17], the relationship between miR396 and *PtrGRFs* was investigated. First, the sequences of *PtrGRFs* and the mature sequences of poplar *miR396b* were uploaded to RNAhybrid [25, 26] to analyze whether *PtrGRFs* are targets of *miR396*. This showed that all of the *PtrGRFs*, except *PtrGRF12b*, have the potential to hybridize with *miR396b* with a minimal free energy hybridization value less than − 33 kcal/mol, suggesting that these *PtrGRFs* could be targets of *miR396* (Fig. 2a). For *PtrGRF12b* and *miR396*, the number of mismatches exceeded the other hybridization pairs and the hybridization energy was − 28 kcal/mol, which exceeded the values observed for most endogenous miRNA targets [27], suggesting that *PtrGRF12b* is not a target of *miR396* (Fig. 2a). Then, we aligned the target sequences of *PtrGRFs* to the mature *miR396b* sequences (Fig. S3). The sequences of *PtrGRF1/2a*-*PtGRF12a* and *miR396* matched perfectly, while a thymine to adenine change in the 3′ terminal of *PtrGRF12b* led to a mismatch, indicating that *PtrGRF12b* is the only *PtrGRF* not targeted by *miR396* (Fig. S3).

In addition, degradome sequencing data [28] were analyzed to identify *miR396* cleavage sites in the *PtrGRFs* (Fig. 2b). As expected, the miR396 cleavage sites of most *PtrGRFs* were found in the degradome data and no such a site was found in the *GRF12b* transcript (Fig. 2b, Table S2), proving the in vivo regulation of the expression of *PtrGRFs* by *miR396* was consistent with the RNAhybrid analysis.

**Fig. 3:**
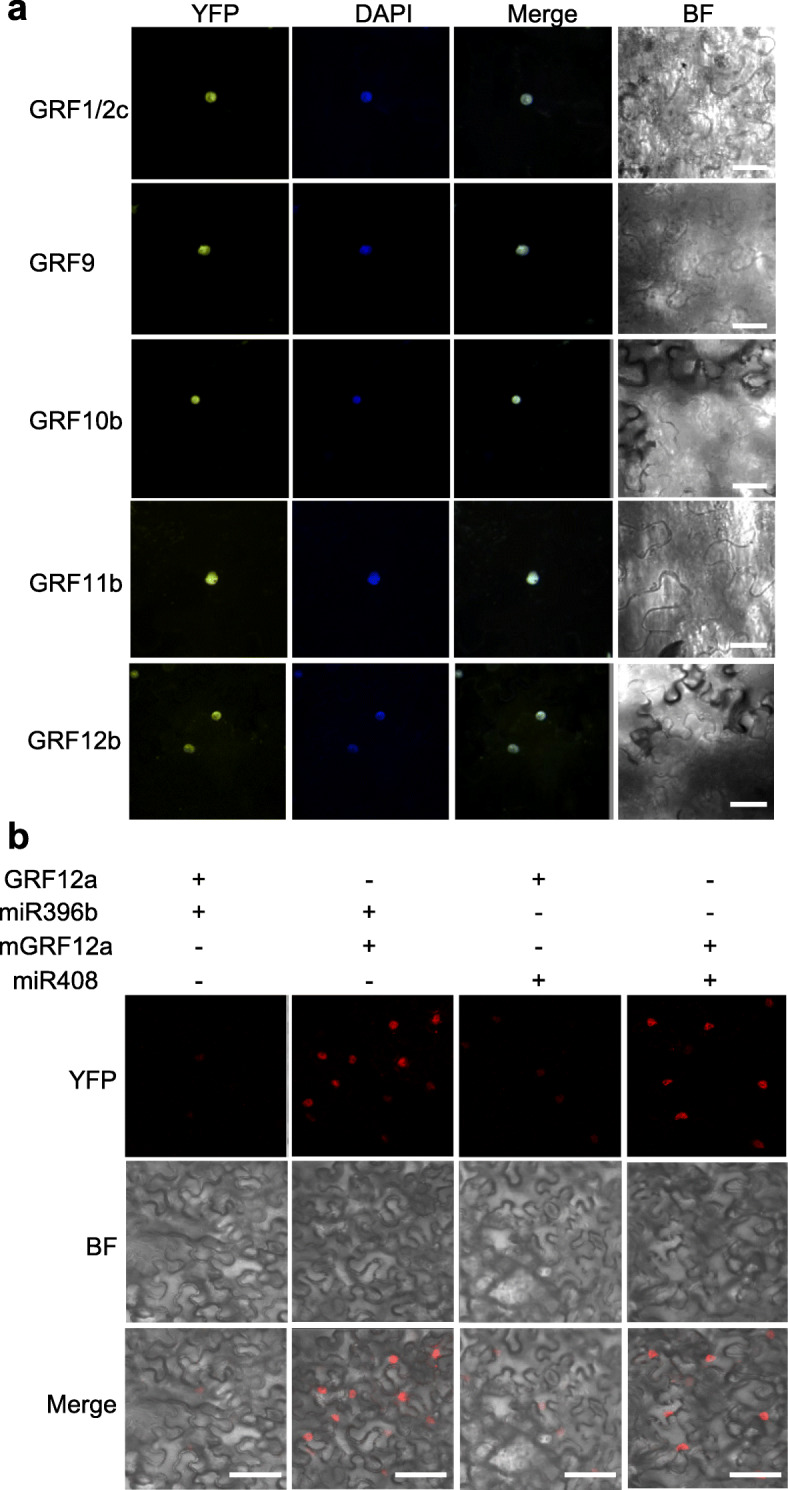
Subcellular localization and miR396 regulation of PtrGRFs. **a** Confocal images of the transient expression of GRF1/2c-YFP, GRF9-YFP, GRF10b-YFP, GRF11b-YFP, and GRF12b-YFP. The GRF1/2c, GRF9, GRF10b, and GRF11b used here all had six nucleotide mutations in their miR396 target sites. Scale bar = 10 μm. **b** GRF12a is targeted by miR396. The fluorescence signal of GRF12a-YFP was faint when co-expressed with miR396b, but the fluorescence signal of mGRF12a-YFP was strong when co-expressed with miR396b. Ptr-miR408 was used as a negative control. Bars = 50 μm

Furthermore, transient expression assay was used to investigate the regulation of poplar *GRFs* by *miR396*. On fusing *PagGRF1/2c*, *PagGRF9*, *PagGRF10b*, *PagGRF11b*, and *PagGRF12b*, genes isolated from poplar 84 K (see Methods), with YFP (Yellow Fluorescent Protein) and expressing them transiently in tobacco leaves, the fluorescence signals of all of the PagGRF-YFP fusion proteins were very weak (Fig. S4), except that of PagGRF12b (Fig. 3a). Considering the functional conservation of plant miRNAs, the weak fluorescence signal may be due to the cleavage of *PagGRFs* by tobacco miR396. To test this, *miR396*-resistant versions of the *GRFs*, which contained six point mutations within the miR396-complementary domain of the *GRF* sequence to increase the number of mismatches without altering the amino acid sequence, were constructed and transiently expressed in tobacco leaves (Fig. S5). As expected, the fluorescence signals of the mPagGRF-YFP fusion proteins were strong and merged with the DAPI signals (Fig. 3a), indicating that miR396 targeted all of the PagGRFs, except PagGRF12b. Furthermore, transient co-expression assays were performed and *PtrmiR408* was used as a negative control to evaluate the regulation of *PagGRF* by *PagmiR396b* (Fig. 3b). Similar to the fluorescent signals of GRF1/2d [previously named GRF15 by Cao et al. (2016)] in our published results [24], the fluorescent signals of GRF12a-YFP were weak when co-expressed with *PtrmiR408*, but were faint and difficult to detect when co-expressed with *PagmiR396b*, indicating that *PagmiR396b* could downregulate the expression of *PagGRF12a*. By contrast, comparable strong fluorescence of mGRF12a-YFP, the mutated version, was detected when co-expressed with *PagmiR396a* or *PtrmiR408*. These results confirmed that *PagmiR396b* could target *PagGRF* directly in vivo.

### Overexpression of *PagGRF6b*, *PagGRF7a*, *PagGRF12a*, and *PagGRF12b* led to diverse changes in leaf size in transgenic poplar

**Fig. 4:**
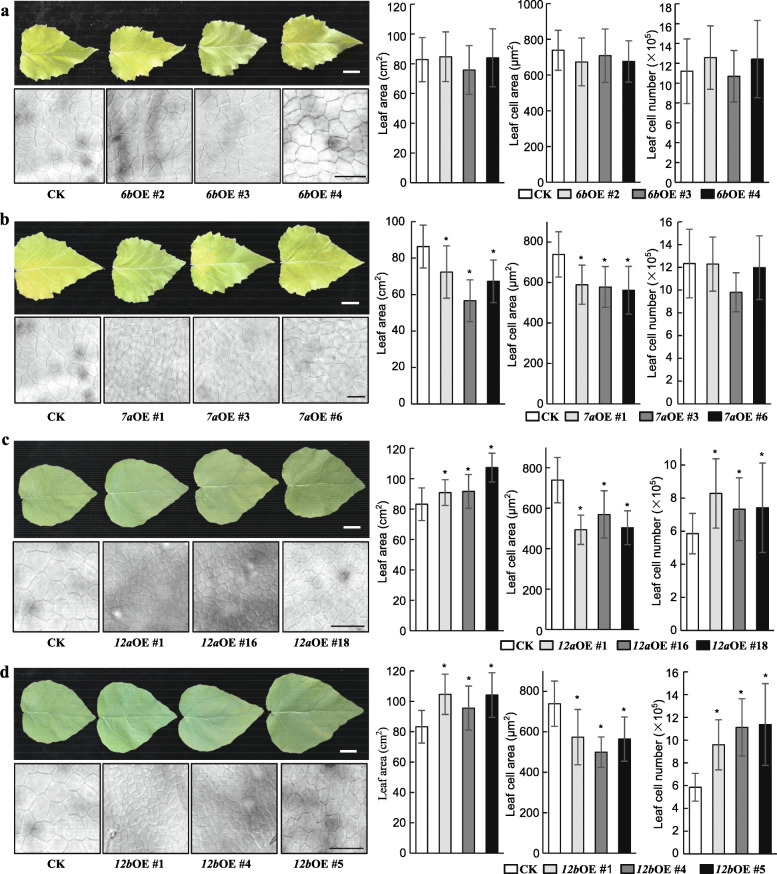
Leaf phenotypes of m*GRF6b* (**a**), m*GRF7a* (**b**), m*GRF12a* (**c**), and *GRF12b* (**d**) overexpression (OE) transgenic plants. Photographs, cell cytology, leaf area, leaf cell area, and calculated leaf cell number of the fifth leaves from 2-month-old m*GRF6b*, m*GRF7a*, m*GRF12a*, and *GRF12b* OE plants are shown. Bar = 2 cm (top) and 50 μm (bottom). Data was presented as means ± SD (*n* = 6–10 for leaf area, *n* = 100–120 for leaf cell area, *n* = 6–10 for leaf cell number). *Ρ < 0.05 determined by Student’s *t*-test

The result in our previous study [24] showed that *GRF1/2a*, *GRF1/2b*, *GRF1/2c*, *GRF1/2d*, *GRF5a*, *GRF5b*, *GRF6b, GRF7a*, *GRF7b*, *GRF8*, *GRF9*, *GRF10a*, *GRF11a*, *GRF11b*, and *GRF12a* were all highly expressed in young leaves, suggesting that these GRFs may have a role in leaf size control. In addition, although its expression was relative low in all tissues, the miR396 independent GRF, *GRF12b*, had higher relative expression in young leaves. Therefore, to investigate the function of poplar GRFs in leaf size control, *PagGRF6b* representing group III, *PagGRF7a* from group IV, and *PagGRF12a* and *PagGRF12b* from group V were chosen to generate transgenic plants for functional characterization (Figs. 1 and 4). The mutated versions of *PagGRF6b*, *PagGRF7a*, and *PagGRF12a*, with synonymous mutations in the miR396 target sites, were used to avoid degradation by miR396 (Fig. S6). Three overexpression (OE) lines each for *mPagGRF6b*, *mPagGRF7a*, *mPagGRF12a*, and *PagGRF12b* with moderately increased expression of the corresponding gene were chosen for further investigation (Fig. S7). The leaf size of the *mPagGRF6b* OE plants did not differ significantly (Fig. 4a), while *mPagGRF7a* OE plants had 26.8% smaller leaves than those of the control (CK) (Fig. 4b). By contrast, *mPagGRF12a* and *PagGRF12b* OE plants had 16.1 and 28.1% larger leaves, respectively, in comparison with CK (Fig. 4c and d).

The leaf epidermis cell area was measured and leaf cell numbers were calculated for *mPagGRF6b*, *mPagGRF7a*, *mPagGRF12a*, and *PagGRF12b* OE plants and compared with the CK. The leaf epidermis cell area of *mPagGRF6b* did not change significantly (Fig. 4a), while it decreased in *mPagGRF7a*, *mPagGRF12a*, and *PagGRF12b* OE plants (Fig. 4b-d). The number of leaf cells in the *mPagGRF6b* and *mPagGRF7a* OE plants did not differ significantly (Fig. 4a and b), but increased significantly in the *mPagGRF12a* and *PagGRF12b* OE plants (Fig. 4c and d).

**Fig. 5:**
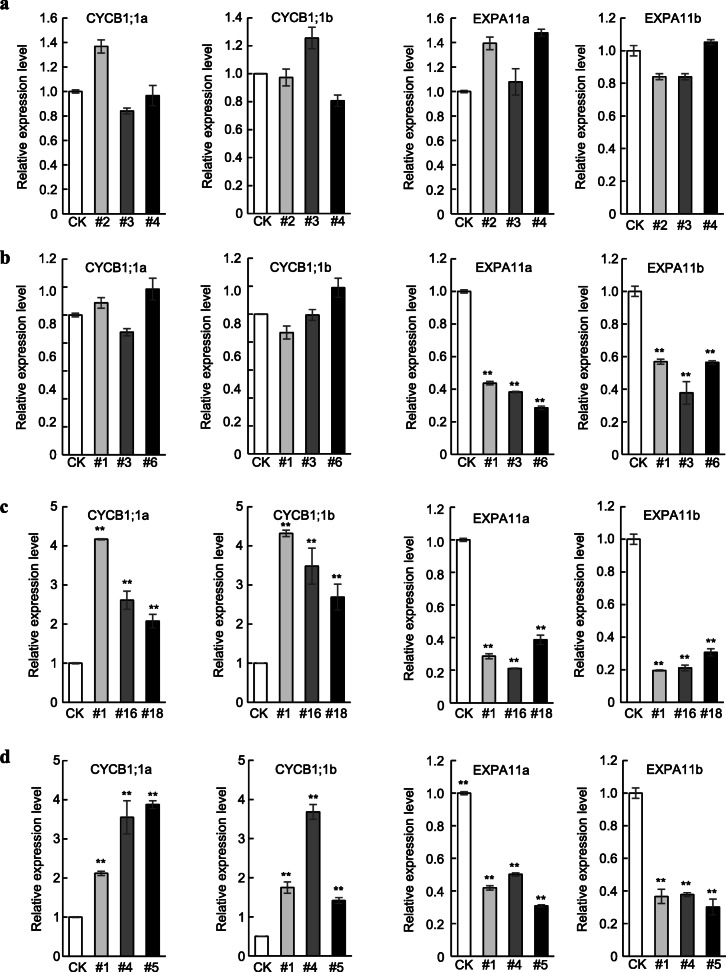
The relative expression of *CYCB1;1* and *EXP11* in leaves of *mGRF6b* (**a**), *mGRF7a* (**b**), *mGRF12a* (**c**), and *GRF12b* (**d**) overexpression (OE) transgenic plants. *CYCB1;1a* and *CYCB1;1b* are the homologs of cell proliferation marker genes in *Arabidopsis*, and *EXP11a* and *EXP11b* are the homologs of cell expansion marker genes in *Arabidopsis*. *Actin* was used as the internal control. Data was presented as means ± SD (*n* = 4–8). *P < 0.05, **P < 0.01 determined by Student’s *t*-test

Furthermore, expression of the cell proliferation marker genes *CYCLINB1;1a* and *CYCLINB1;1b* and cell expansion marker genes *EXPA11a* and *EXPA11b* (Zhou et al. 2019) was examined in the fifth leaves from *mPagGRF6b*, *mPagGRF7a*, *mPagGRF12a*, and *PagGRF12b* OE plants. Consistent with our observations, expression of *CYCLINB1;1a* and *CYCLINB1;1b* was unaltered in *mPagGRF6b* and *mPagGRF7a* OE plants, but upregulated in the *mPagGRF12a* and *PagGRF12b* OE plants (Fig. 5, Fig. S8). Meanwhile, the expression of *EXPA11a* and *EXPA11b* did not change much in the *mPagGRF6b* OE plants, but was downregulated significantly in *mPagGRF7a*, *mPagGRF12a*, and *PagGRF12b* OE plants (Fig. 5, Fig. S8).

These results indicate that *PagGRF6b* has no function in leaf size control; *PagGRF7a* functions as a negative regulator of leaf size, mainly by regulating cell expansion; and *PagGRF12a* and *PagGRF12b* positively regulate leaf size through both cell proliferation and cell expansion, but mainly through cell proliferation.

## Discussion

The expansion of GRFs in *Populus* and their functional diversification in leaf development have drawn our attention. We have re-grouped the 19 GRFs identified in the *P. trichocarpa* genome into six groups according to their phylogenetic relationships with their *Arabidopsis* counterparts and renamed these poplar GRFs based on orthology. This facilitates the comparison of the evolution and functional diversity of GRF members in *Populus* and *Arabidopsis*. We found that as a result of the divergence of *GRF* sequences in *Populus*, one of the 19 *PtrGRFs*, *PtrGRF12b*, was not targeted by miR396 and that *PagGRF6b*, *PagGRF7a*, *PagGRF12a*, and *PagGRF12b* worked differently in leaf size control.

Previously, we reported that PagGRF15 (which named as PagGRF1/2d in this study) could work as a positive regulator on leaf size through mainly regulating cell expansion [24]. Here, we found that PagGRF7a acts distinctly as a negative regulator on leaf size while PagGRF6b has no effect on leaf development, though PagGRF12a and PagGRF12b are similar to PagGRF1/2d functioning as positive regulators, indicating that different members of poplar GRFs have distinct roles in leaf size control. In addition, we found that PagGRF7a regulates leaf size through negatively affecting cell expansion while PagGRF12a and PagGRF12b though positively affecting cell proliferation and negatively regulating cell expansion. All these GRFs exhibit differences with PagGRF1/2d that positively affects cell expansion. The unexpected diversified regulation on leaf size control by various poplar GRFs provides more and additional information than our previous report about PagGRF1/2d.

*miR396* regulates the expression of *GRFs* through direct cleavage of complementary sequences in the *GRF* genes [18]. Here, we found that 18 *PtrGRFs* were regulated by *miR396*, with *PtrGRF12b* the only exception, based on sequence comparison, the cleavage sites of transcripts, and in vivo miRNA-target analysis. In comparison, *miR396* did not target two GRFs in *A. thaliana*, *AtGRF5* and *AtGRF6* [29, 30]. *PtrGRF12b* belongs to group V, while *AtGRF5* and *AtGRF6* belong to group III, suggesting that the miR396 regulation pattern of *GRFs* has different features in the two species. It was recently reported that AtGRF5 plays roles in chloroplast development, nitrogen signaling, and senescence, apart from its function in leaf development [31]. Therefore, the loss and gain of miR396 regulation of GRFs may cause functional shifts in their roles in plant growth and development. Further studies are needed to investigate whether PtrGRF12b has functions in addition to those of other miR396-regulated GRFs.

Additionally, our study also suggests the importance of the regulation of GRF genes by miR396 in poplar, which is found in *Arabidopsis* [18, 21, 32]. Firstly, the miR396 regulation on GRFs using miR396-resistant version have been tested through transient expression assays in order to obtain the overexpression of GRFs. The fluorescence signals of the cells expressing *PagGRF6b*-YFP, *PagGRF7a*-YFP and *PagGRF12a*-YFP were faint and hardly detected, while the signals expressed their miR396-resistant version were strong, exampled as *PagGRF12a*-YFP in Fig. 3, which indicated that these GRFs were regulated by the existing miR396 in cells. Secondly, although PagGRF12a and PagGRF15 (PagGRF1/2d in this study) [24] are positive regulators on leaf size, the *PagmiR396b* OE plants showed a phenotype of smaller leaves [24], suggesting the “epistasis” effect of miR396b on the regulation of leaf size. Therefore, the interaction of miR396 and GRFs is important for leaf development.

GRFs are important regulators of leaf development [3, 4], and we found the functional divergence of GRFs in this study. PagGRF7a is a negative regulator, while PagGRF12a and PagGRF12b are positive regulators of leaf size, and PagGRF6b has no effect on leaf size. In *Arabidopsis*, AtGRF1, AtGRF2, AtGRF3, AtGRF5, and AtGRF7 all function as positive regulators of leaf size [15, 18, 21, 32], while only AtGRF9 functions as a negative regulator of leaf size [33]. Therefore, GRFs from poplar and *Arabidopsis* show diverse regulation of leaf size and their functions need to be assessed individually. In addition, it is interested that poplar GRF12a and GRF12b, like rice and maize GRF10 [23, 30], have truncated C-terminal end. It was proposed that overexpression of *ZmGRF10* may break the homeostasis of distinct GRF/GIF complexes and result in the altered representation of other GRF/GIF complex to affect leaf growth [23], whether poplar GRF12a and GRF12b also work in this way needs to be investigated.

We also noticed that poplar and *Arabidopsis* GRFs classified in the same group could function in different ways in leaf size control. For instance, PagGRF6b from Group III has no effect on leaf size, while AtGRF5 from Group III is a positive regulator of leaf size [15]. *PagGRF7a* from Group IV functions as a negative regulator, while AtGRF7 from the same group functions as a positive regulator of leaf size [32]. Similarly, *PagGRF12a* and *PagGRF12b* from Group V work as positive regulators, while *AtGRF9* from Group V is a negative regulator of leaf size [33]. In comparison, although PagGRF1/2d from Group I is similar to AtGRF1 and AtGRF2 from Group I [18] and all three function as positive regulators of leaf size [24], PagGRF1/2d functions mainly by regulating cell expansion, while AtGRF1 and AtGRF2 function mainly by regulating cell proliferation. Therefore, the ways in which GRFs control leaf size in poplar cannot be simply inferred from their orthologs in *Arabidopsis*.

Previously, we reported that PagGRF1/2d regulated leaf size mainly by regulating cell expansion in poplar [24], which is different from all reported *Arabidopsis* GRFs, including AtGRF1, AtGRF2, AtGRF5, AtGRF7, and AtGRF9, which mainly act by regulating cell proliferation [15, 18, 21, 32, 33]. In this study, we found that PagGRF12a and PagGRF12b are involved in leaf size control mainly through regulating cell proliferation, while PagGRF7a and PagGRF1/2d negatively or positively, respectively, regulate leaf size mainly by regulating cell expansion. Therefore, the underlying mechanisms by which GRFs regulate leaf size are more diverse in poplar than in *Arabidopsis*. Leaf size is important for biomass production in woody plants [34] and should be under tight control. Poplar has more than twice the number of GRFs than *Arabidopsis* (19 vs. 9), so the diverse regulation in leaf size of these GRFs in poplar will facilitate the specific and coordinated regulation of leaf development through fine-tuning of cell proliferation and expansion.

## Conclusions

In conclusion, we analyzed the phylogenetic relationship of GRF genes in *Populus* with their counterparts in *Arabidopsis* and functionally characterized PagGRF6b, PagGRF7a, PagGRF12a, and PagGRF12b, which work differently in leaf size control in transgenic poplar. This diversity may facilitate the specific, coordinated regulation of poplar leaf development through fine adjustment of cell proliferation and expansion. Our findings provide an abundant resource for genetic engineering leaf size in trees.

## Methods

### Phylogenetic tree construction

*Populus GRF* gene sequences were downloaded from the Poplar Genome Database (http://www.phytozome.net/poplar.php, release 3.0). All sequences were confirmed according to the annotation of the QLQ and WRC domains. WoLF PSORT (http://wolfpsort.org) was used to predict the protein subcellular localization. The pI and molecular weight were estimated using Lasergene. The full-length protein sequences were aligned using ClustalX2 (ver. 2.1) [35]. A neighbor-joining phylogenetic tree was constructed using MEGA (v5.0) with the bootstrap method (1000 bootstrap replicates, Poisson model, uniform rates, and pairwise deletion) [36]. Functional motifs or domains of PtrGRF sequences were analyzed using the reported FFD, GPL, and TQL motifs [8] as queries to find the corresponding sequences.

### Degradome sequencing

The degradome data was from our previous study [28]. In brief, the degradome libraries of *P. tomentosa* were constructed from the poly(A) tail-containing fraction of total RNA samples pooled from the regenerating tissues after girdling to identify target genes of miRNAs. Then, data were analyzed using the CleaveLand pipeline and psRNATarget (http://plantgrn.noble.org/psRNATarget/) to predict the targets of miRNAs against the transcript sequences of *P. trichocarpa* genome (V3.0).

### Transient expression assay

The transient expression assay was conducted according to our previous report [24]. *GRF1/2c*, *GRF9*, *GRF10b*, *GRF11b*, and *GRF12b* were cloned from the hybrid poplar clone 84 K (*Populus alba* × *P. glandulosa*, *Pag*) reserved by State Key Laboratory of Tree Genetics and Breeding, Chinese Academy of Forestry. pEarleyGate 101 vector was used to generate the PagGRF-YFP construct, while the pMDC32 vector was used to overexpress *PagmiR396b* and *PtrmiR408*. The various construct combinations were introduced into 1-month-old *Nicotiana benthamiana* (reserved by State Key Laboratory of Tree Genetics and Breeding, Chinese Academy of Forestry) leaves through *Agrobacterium*-mediated infiltration. Fluorescence signals were probed using LSM 510 AX70 (Zeiss).

### Plant transformation

Plant transformation was done as previously reported [24]. *GRF6b*, *GRF7a*, *GRF12a*, and *GRF12b* were cloned from 84 K using the primers listed in Table S3. pMDC32 vector was used to overexpress *PagGRF6b*, *PagGRF7a*, *PagGRF12a*, and *PagGRF12b*. All vectors were transformed into 84 K leaf discs via *Agrobacterium*-mediated transformation. Tissue-cultured plants were grown under long-day conditions (16 h light/8 h dark). Transgenic plants were confirmed by examining the expression of the corresponding genes.

### Leaf phenotyping

Leaf phenotyping was performed as described in our previous study [24]. Briefly, the first completely uncurled leaf was defined as the first leaf. The fifth leaves of OE and CK plants were detached, fixed with FAA (formaldehyde: acetic acid: 96% alcohol: water; 10:5:50:35), cleared with chloral solution (200 g chloral hydrate, 20 g glycerol, and 50 mL dH_2_O), and surveyed using a confocal Zeiss LSM 510 AX70 microscope. The cell number in the lower epidermis was calculated by dividing the leaf area by the area of epidermal cells. At least six leaves were used for the leaf area measurements and more than 100 epidermal cells in each leaf were used for cell area measurements.

### RNA isolation and quantitative RT-PCR

The expression of *CYCLINB1;1a*, *CYCLINB1;1b*, *EXPA11a*, and *EXPA11b* in OE and CK plants was analyzed using quantitative RT-PCR (qRT-PCR) according to our previous study [24]. Briefly, the fifth leaves were collected from 2-month-old OE and CK plants and total RNA was extracted using the easy-spin plus RNeasy Plant Mini Kit (Aidlab, Beijing, China). First-strand cDNA was synthesized using the SuperScript III reverse transcription kit (TaKaRa, Dalian, China) and oligo dT primers. All primer sequences are listed in Table S3. Real-time PCR was conducted on a LightCycler 480 (Roche, Basel, Switzerland) using SYBR Premix Ex Taq™ Kit (TaKaRa, Dalian, China). *Actin* and *UBQ* were used as internal controls.

## Supplementary information

**Additional file 1: Figure S1.** QLQ, WRC domains and FFD, TQL and GPL motifs of *Populus* GRFs.

**Additional file 2: Figure S2.** Phylogenetic relationship of the *GRF* genes from *P. trichocarpa* (Ptr), *A.thaliana* (At), *O. sativa* (Os), *S. bicolor* (Sb), *Z. mays* (Zm), *V. vinifera* (Vv), *G. max* (Gm) and *P. patens* (Pp).

**Additional file 3: Figure S3.** Diagram of the complemental sites of miR396b to PtrGRFs.

**Additional file 4: Figure S4.** Subcellular localization of GRF1/2c, GRF9, GRF10b, and GRF11b. Bars = 20 μm.

**Additional file 5: Figure S5.** Diagram showed the introduction of synonymous mutations of the miR396 target sites in the mGRF1/2c, mGRF9, mGRF10b, mGRF11b.

**Additional file 6: Figure S6.** Diagram showed the introduction of synonymous mutations of the miR396 target sites in the mGRF6b, mGRF7a, and mGRF12a.

**Additional file 7: Figure S7.** Photograph of mGRF6b, mGRF7a, mGRF12a and GRF12b OE plants and the expression level of mGRF6b, mGRF7a, mGRF12a and GRF12b in mGRF6b, mGRF7a, mGRF12a and GRF12b OE plants. Data was presented as means ± SD (*n* = 4–8). **P < 0.01 determined by Student’s *t*-test.

**Additional file 8: Figure S8.** The relative expression of *CYCB1;1* and *EXP11* in leaves of *mGRF6b* (a), *mGRF7a* (b), *mGRF12a* (c), and *GRF12b* (d) overexpression (OE) transgenic plants. UBQ was used as internal control. Data was presented as means ± SD (*n* = 4–8). *P < 0.05, **P < 0.01 determined by Student’s *t*-test.

**Additional file 9: Table S1.** Complete gene information of PtrGRFs and AtGRFs.

**Additional file 10: Table S2.** The cleavage site of PtrGRFs by miR396 in the degradome data from Tang et al. (2016).

**Additional file 11: Table S3.** Primers used in gene cloning and qRT-PCR analysis.

## Data Availability

All data generated or analyzed during this study are included in this article (and its supplementary information files) or are available from the corresponding author on reasonable request. Protein sequences have been deposited in GenBank (PagGRF6b, MW014326; PagGRF7a, MW015994; PagGRF9, MW015997; PagGRF10b, MW015995; PagGRF11b, MW015996; PagGRF12a, MW014327; PagGRF12b, MW014328).

## Abbreviations

GRF: Growth-regulating factor
QLQ: Glu-Leu-Glu
WRC: Trp-Arg-Cys
*Pag*: *Populus alba* × *P. glandulosa*
*At*: *Arabidopsis thaliana*
*Bn*: *Brassica napus*
*Br*: *Brassica rapa*
*Zm*: *Zea mays*
*Ptr*: *Populus trichocarpa*
YFP: Yellow Fluorescent Protein
OE: Overexpression
qRT-PCR: quantitative real time polymerase chain reaction

## References

[1] Powell AE, Lenhard M (2012). Control of organ size in plants. Curr Biol.

[2] Hepworth J, Lenhard M (2014). Regulation of plant lateral-organ growth by modulating cell number and size. Curr Opin Plant Biol.

[3] Hoe Kim J, Tsukaya H (2015). Regulation of plant growth and development by the GROWTH-REGULATING FACTOR and GRF-INTERACTING FACTOR duo. J Exp Bot.

[4] Omidbakhshfard MA, Proost S, Fujikura U, Mueller-Roeber B (2015). Growth-regulating factors (GRFs): a small transcription factor family with important functions in plant biology. Mol Plant.

[5] van der Knaap E, Kim JH, Kende H (2000). A novel gibberellin-induced gene from rice and its potential regulatory role in stem growth. Plant Physiol.

[6] Kim JH, Choi D, Kende H (2003). The AtGRF family of putative transcription factors is involved in leaf and cotyledon growth in *Arabidopsis*. Plant J.

[7] Choi D, Kim JH, Kende H (2004). Whole genome analysis of the OsGRF gene family encoding plant-specific putative transcription activators in rice (*Oryza sativa* L.). Plant Cell Physiol.

[8] Zhang DF, Jia GQ, Zhang TF, Dai JR, Li JS, Wang SC (2008). Isolation and characterization of genes encoding GRF transcription factors and GIF transcriptional coactivators in maize (*Zea mays* L.). Plant Sci.

[9] Bazin J, Khan GA, Combier JP, Bustos-Sanmamed P, Debernardi JM, Rodriguez R, Sorin C, Palatnik J, Hartmann C, Crespi M, Lelandais-Brière C (2013). miR396 affects mycorrhization and root meristem activity in the legume *Medicago truncatula*. Plant J.

[10] Wang F, Qiu N, Ding Q, Li J, Zhang Y, Li H, Gao J (2014). Genome-wide identification and analysis of the growth-regulating factor family in Chinese cabbage (*Brassica rapa* L. ssp. pekinensis). BMC Genomics.

[11] Cao Y, Han Y, Jin Q, Lin Y, Cai Y (2016). Comparative genomic analysis of the GRF aenes in Chinese pear (*Pyrus bretschneideri* Rehd), poplar (*Populous*), grape (*Vitis vinifera*), *Arabidopsis* and Rice (*Oryza sativa*). Front Plant Sci.

[12] Liu X, Guo LX, Jin LF, Liu YZ, Liu T, Fan YH, Peng SA (2016). Identification and transcript profiles of citrus growth-regulating factor genes involved in the regulation of leaf and fruit development. Mol Biol Rep.

[13] Khatun K, Robin AHK, Park JI, Nath UK, Kim CK, Lim KB, Nou IS, Chung MY. Molecular characterization and expression profiling of tomato GRF transcription factor family genes in response to abiotic stresses and phytohormones. Int J Mol Sci. 2017;18(5):1056. 10.3390/ijms18051056 PMC5454968 28505092

[14] Zhang J, Li Z, Jin J, Xie X, Zhang H, Chen Q, Luo Z, Yang J (2018). Genome-wide identification and analysis of the growth-regulating factor family in tobacco (*Nicotiana tabacum*). Gene..

[15] Horiguchi G, Kim GT, Tsukaya H (2005). The transcription factor AtGRF5 and the transcription coactivator AN3 regulate cell proliferation in leaf primordia of *Arabidopsis thaliana*. Plant J.

[16] Kim JS, Mizoi J, Kidokoro S, Maruyama K, Nakajima J, Nakashima K, Mitsuda N, Takiguchi Y, Ohme-Takagi M, Kondou Y, Yoshizumi T, Matsui M, Shinozaki K, Yamaguchi-Shinozaki K (2012). *Arabidopsis* growth-regulating factor7 functions as a transcriptional repressor of abscisic acid- and osmotic stress-responsive genes, including DREB2A. Plant Cell.

[17] Debernardi JM, Rodriguez RE, Mecchia MA, Palatnik JF (2012). Functional specialization of the plant miR396 regulatory network through distinct microRNA-target interactions. PLoS Genet.

[18] Rodriguez RE, Mecchia MA, Debernardi JM, Schommer C, Weigel D, Palatnik JF (2010). Control of cell proliferation in Arabidopsis thaliana by microRNA miR396. Development.

[19] Wang L, Gu X, Xu D, Wang W, Wang H, Zeng M, Chang Z, Huang H, Cui X (2011). miR396-targeted AtGRF transcription factors are required for coordination of cell division and differentiation during leaf development in *Arabidopsis*. J Exp Bot.

[20] Liu J, Hua W, Yang HL, Zhan GM, Li RJ, Deng LB, Wang XF, Liu GH, Wang HZ (2012). The BnGRF2 gene (GRF2-like gene from *Brassica napus*) enhances seed oil production through regulating cell number and plant photosynthesis. J Exp Bot.

[21] Debernardi JM, Mecchia MA, Vercruyssen L, Smaczniak C, Kaufmann K, Inze D, Rodriguez RE, Palatnik JF (2014). Post-transcriptional control of GRF transcription factors by microRNA miR396 and GIF co-activator affects leaf size and longevity. Plant J.

[22] Nelissen H, Eeckhout D, Demuynck K, Persiau G, Walton A, van Bel M, Vervoort M, Candaele J, De Block J, Aesaert S, Van Lijsebettens M, Goormachtig S, Vandepoele K, Van Leene J, Muszynski M, Gevaert K, Inzé D, De Jaeger G (2015). Dynamic changes in ANGUSTIFOLIA3 complex composition reveal a growth regulatory mechanism in the maize leaf. Plant Cell.

[23] Wu L, Zhang D, Xue M, Qian J, He Y, Wang S (2014). Overexpression of the maize GRF10, an endogenous truncated growth-regulating factor protein, leads to reduction in leaf size and plant height. J Integr Plant Biol.

[24] Zhou H, Song X, Wei K, Zhao Y, Jiang C, Wang J, Tang F, Lu M (2019). Growth-regulating factor 15 is required for leaf size control in *Populus*. Tree Physiol.

[25] Rehmsmeier M, Steffen P, Hochsmann M, Giegerich R (2004). Fast and effective prediction of microRNA/target duplexes. RNA..

[26] Kruger J, Rehmsmeier M (2006). RNAhybrid. microRNA target prediction easy, fast and flexible. Nucleic Acids Res.

[27] Schwab R, Palatnik JF, Riester M, Schommer C, Schmid M, Weigel D (2005). Specific effects of microRNAs on the plant transcriptome. Dev Cell.

[28] Tang F, Wei H, Zhao S, Wang L, Zheng H, Lu M (2016). Identification of microRNAs involved in regeneration of the secondary vascular system in *Populus tomentosa* Carr. Front Plant Sci.

[29] Jones-Rhoades MW, Bartel DP, Bartel B (2006). MicroRNAS and their regulatory roles in plants. Annu Rev Plant Biol.

[30] Liu H, Guo S, Xu Y, Li C, Zhang Z, Zhang D, Xu S, Zhang C, Chong K (2014). OsmiR396d-regulated OsGRFs function in floral organogenesis in rice through binding to their targets OsJMJ706 and OsCR4. Plant Physiol.

[31] Vercruyssen L, Tognetti VB, Gonzalez N, Van Dingenen J, De Milde L, Bielach A, De Rycke R, Van Breusegem F, Inze D (2015). GROWTH REGULATING FACTOR5 stimulates *Arabidopsis* chloroplast division, photosynthesis, and leaf longevity. Plant Physiol.

[32] Liang G, He H, Li Y, Wang F, Yu D (2014). Molecular mechanism of microRNA396 mediating pistil development in *arabidopsis*. Plant Physiol.

[33] Omidbakhshfard MA, Fujikura U, Olas JJ, Xue GP, Balazadeh S, Mueller-Roeber B (2018). GROWTH-REGULATING FACTOR 9 negatively regulates *arabidopsis* leaf growth by controlling ORG3 and restricting cell proliferation in leaf primordia. PLoS Genet.

[34] Ridge CR, Hinckley TM, Stettler RF, Van Volkenburgh E (1986). Leaf growth characteristics of fast-growing poplar hybrids *Populus trichocarpa* x *P. deltoides*. Tree Physiol.

[35] Larkin MA, Blackshields G, Brown NP, Chenna R, McGettigan PA, McWilliam H, Valentin F, Wallace IM, Wilm A, Lopez R, Thompson JD, Gibson TJ, Higgins DG (2007). Clustal W and Clustal X version 2.0. Bioinformatics..

[36] Jones DT, Taylor WR, Thornton JM (1992). The rapid generation of mutation data matrices from protein sequences. Comput Appl Biosci.

