# Supplementary figures and images for: Characterization of poplar growth-regulating factors and analysis of their function in leaf size control
Source: BMC Plant Biol. 2020 Nov 5;20:509. doi: 10.1186/s12870-020-02699-4 (PMC7643314; doi:10.1186/s12870-020-02699-4)

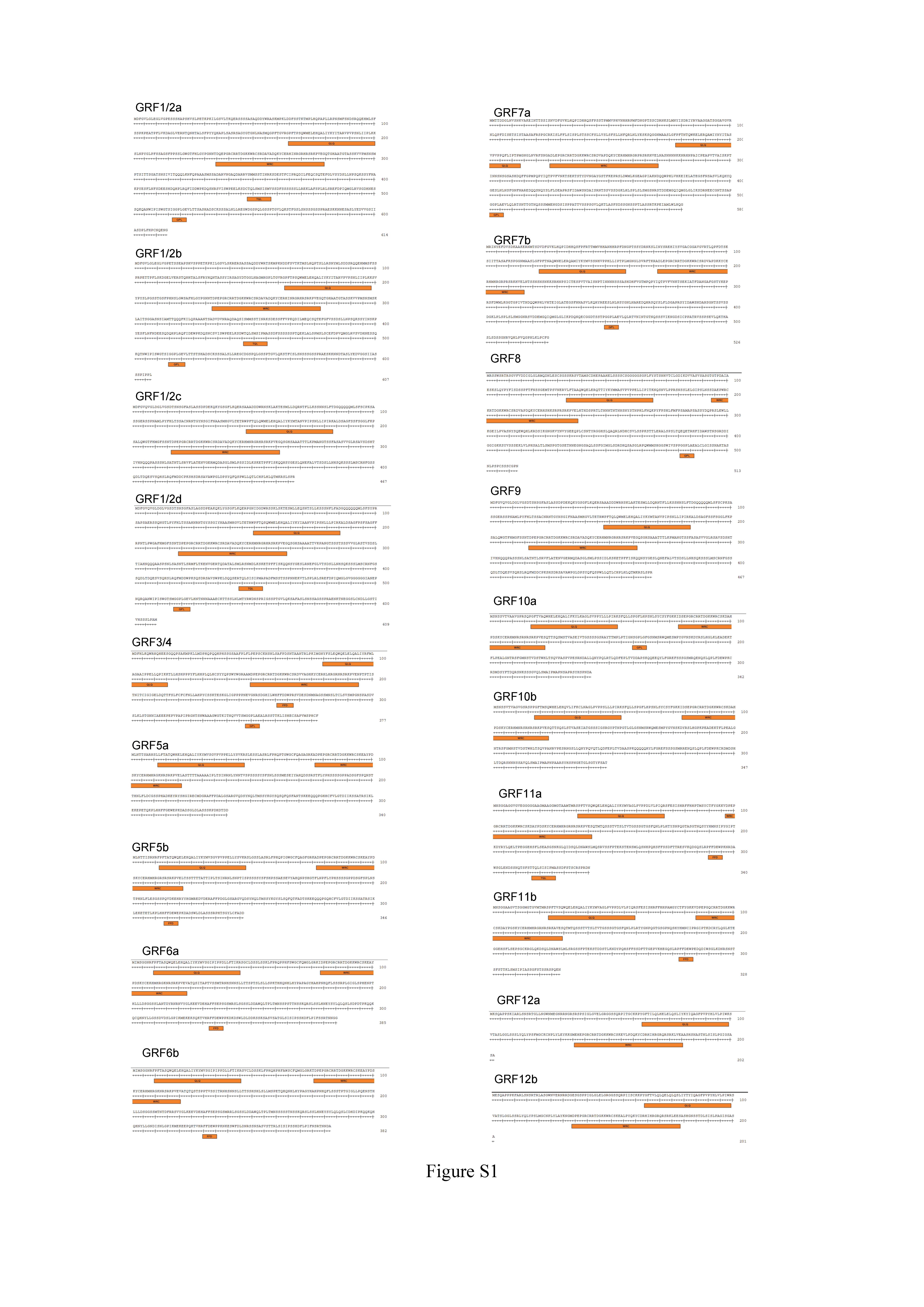

Supplement: Supplementary file 1 — Additional file 1: Figure S1. QLQ, WRC domains and FFD, TQL and GPL motifs of Populus GRFs. [file 12870_2020_2699_MOESM1_ESM.tif]

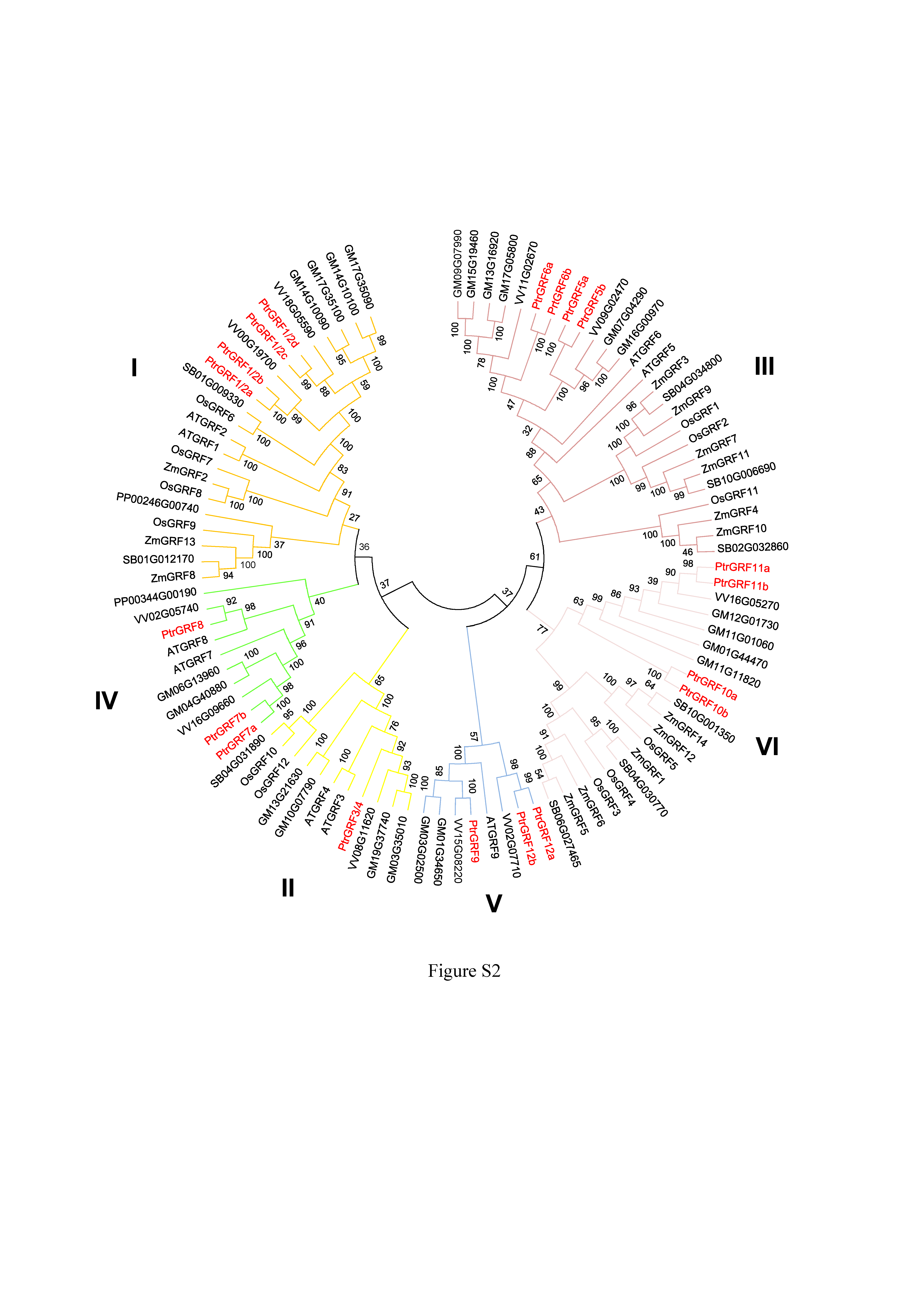

Supplement: Supplementary file 2 — Additional file 2: Figure S2. Phylogenetic relationship of the GRF genes from P. trichocarpa (Ptr), A.thaliana (At), O. sativa (Os), S. bicolor (Sb), Z. mays (Zm), V. vinifera (Vv), G. max (Gm) and P. patens (Pp). [file 12870_2020_2699_MOESM2_ESM.tif]

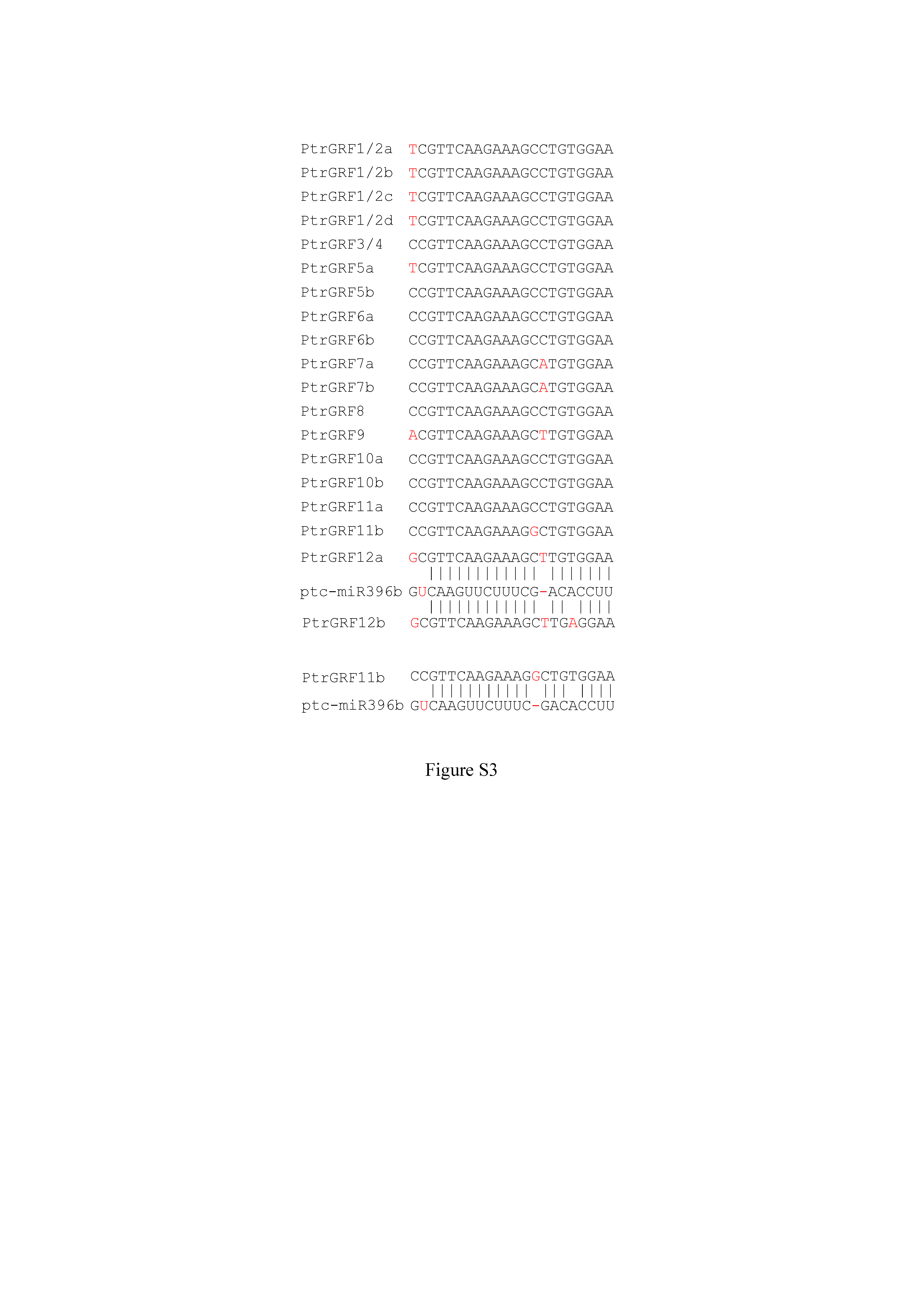

Supplement: Supplementary file 3 — Additional file 3: Figure S3. Diagram of the complemental sites of miR396b to PtrGRFs. [file 12870_2020_2699_MOESM3_ESM.tif]

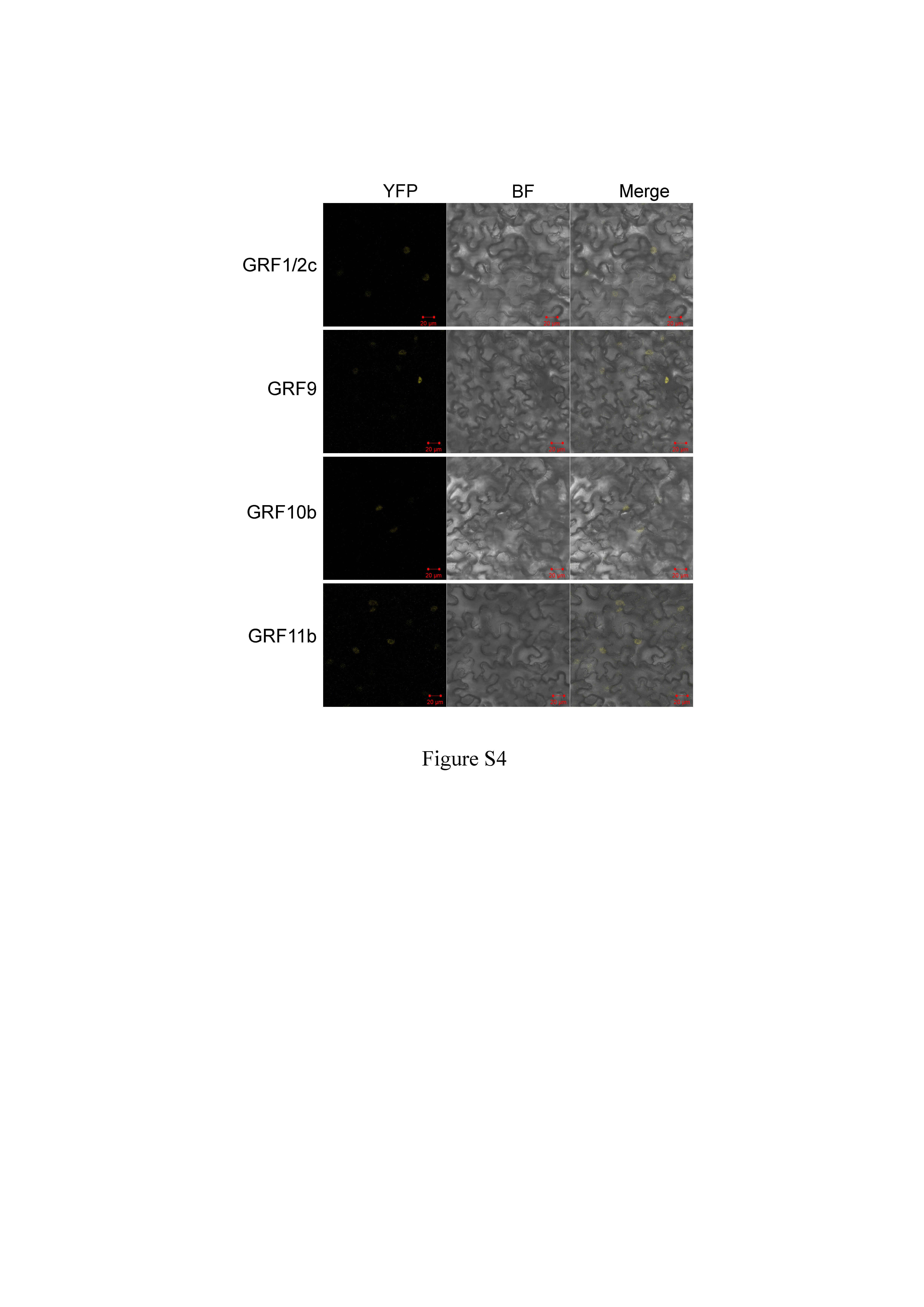

Supplement: Supplementary file 4 — Additional file 4: Figure S4. Subcellular localization of GRF1/2c, GRF9, GRF10b, and GRF11b. Bars = 20 μm. [file 12870_2020_2699_MOESM4_ESM.tif]

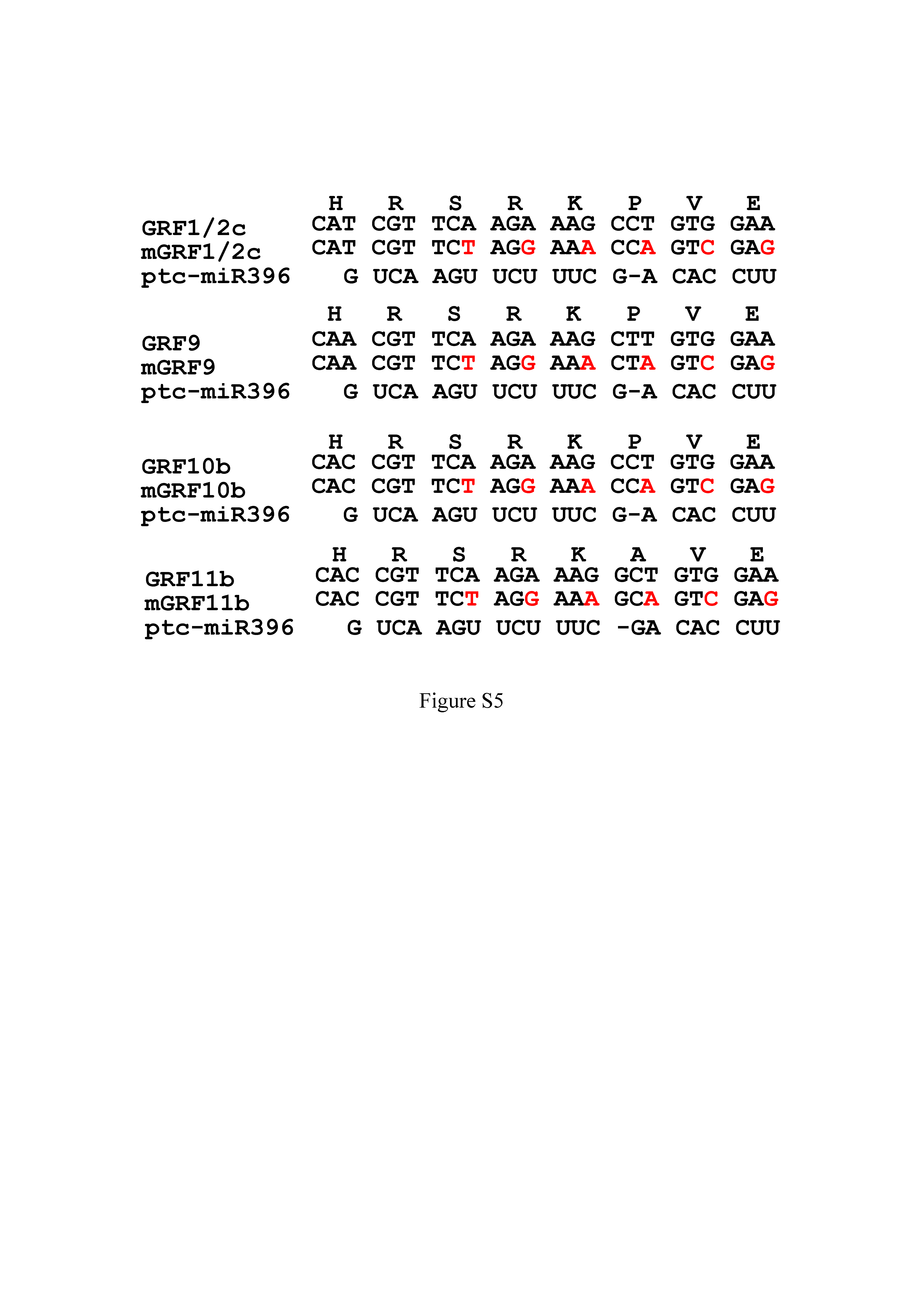

Supplement: Supplementary file 5 — Additional file 5: Figure S5. Diagram showed the introduction of synonymous mutations of the miR396 target sites in the mGRF1/2c, mGRF9, mGRF10b, mGRF11b. [file 12870_2020_2699_MOESM5_ESM.tif]

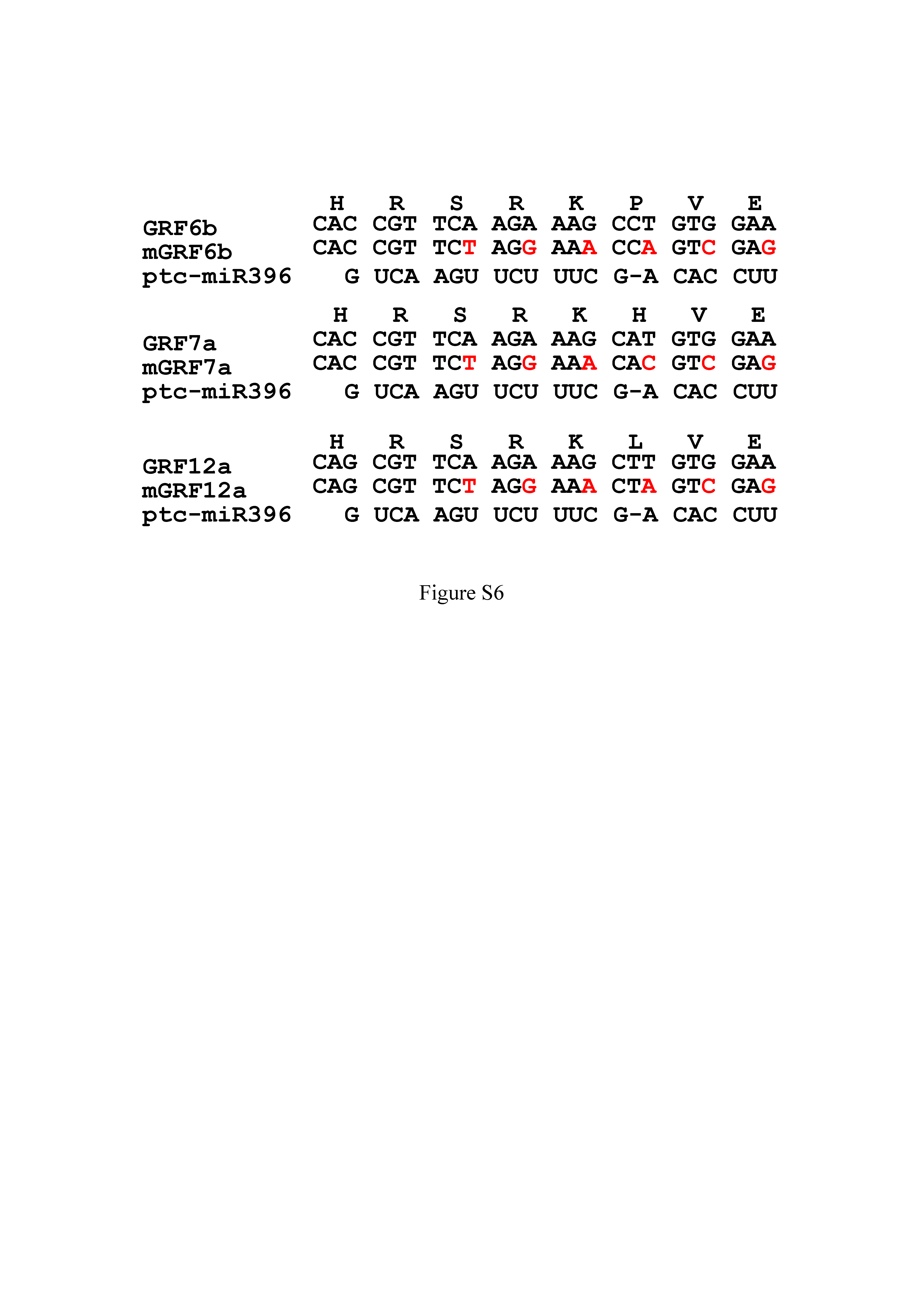

Supplement: Supplementary file 6 — Additional file 6: Figure S6. Diagram showed the introduction of synonymous mutations of the miR396 target sites in the mGRF6b, mGRF7a, and mGRF12a. [file 12870_2020_2699_MOESM6_ESM.tif]

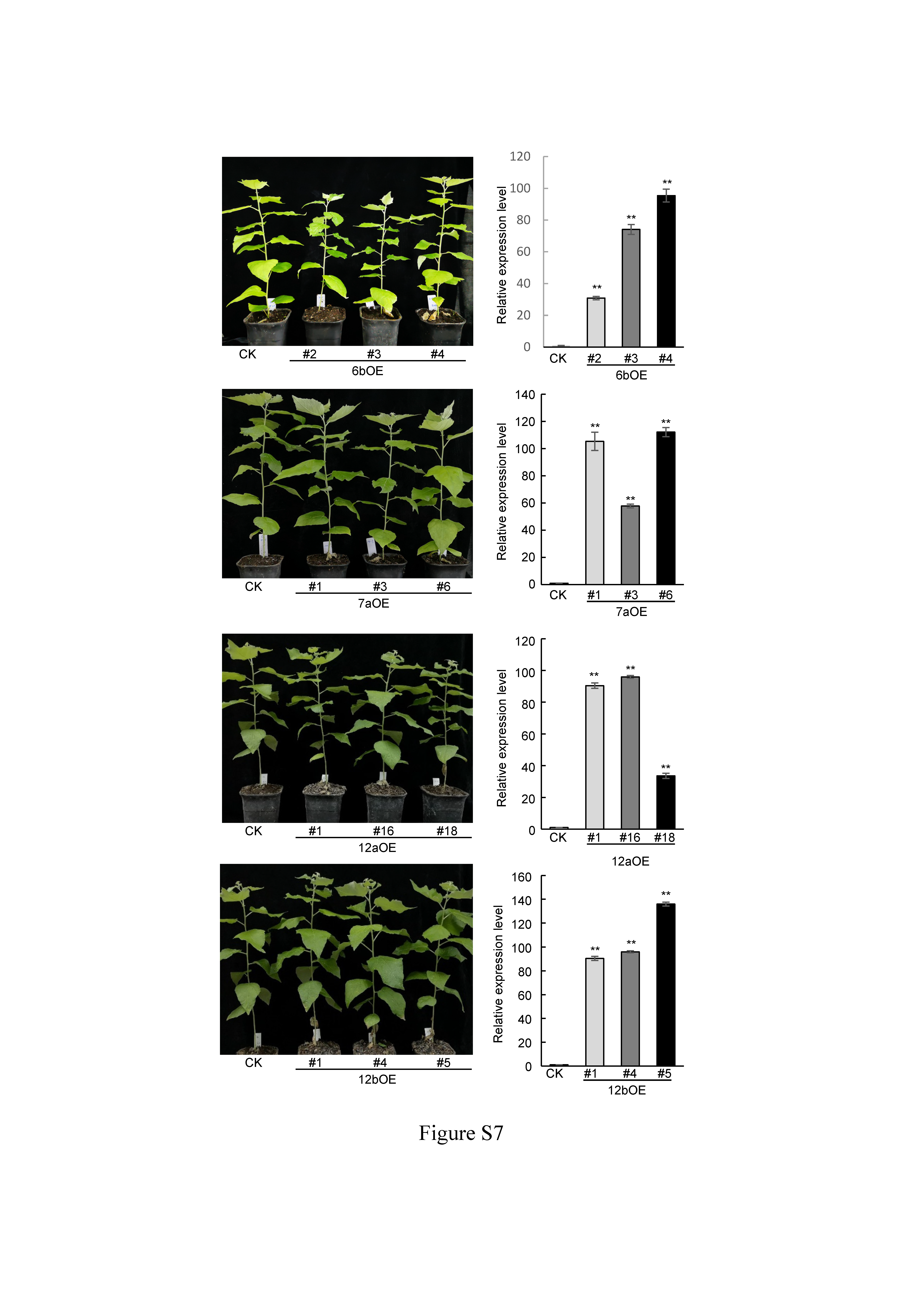

Supplement: Supplementary file 7 — Additional file 7: Figure S7. Photograph of mGRF6b, mGRF7a, mGRF12a and GRF12b OE plants and the expression level of mGRF6b, mGRF7a, mGRF12a and GRF12b in mGRF6b, mGRF7a, mGRF12a and GRF12b OE plants. Data was presented as means ± SD (n = 4–8). **P < 0.01 determined by Student’s t-test. [file 12870_2020_2699_MOESM7_ESM.tif]

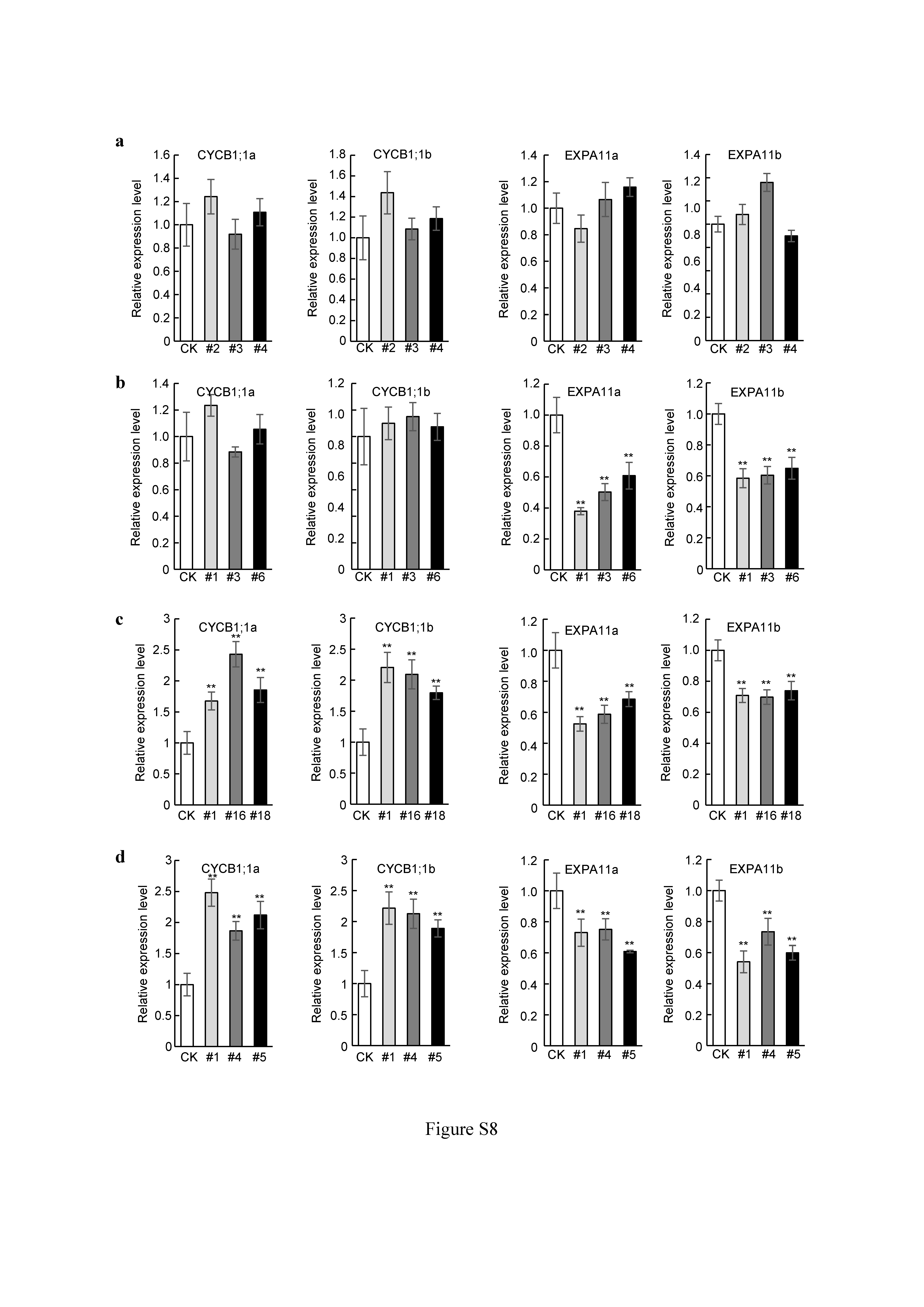

Supplement: Supplementary file 8 — Additional file 8: Figure S8. The relative expression of CYCB1;1 and EXP11 in leaves of mGRF6b (a), mGRF7a (b), mGRF12a (c), and GRF12b (d) overexpression (OE) transgenic plants. UBQ was used as internal control. Data was presented as means ± SD (n = 4–8). *P < 0.05, **P < 0.01 determined by Student’s t-test. [file 12870_2020_2699_MOESM8_ESM.tif]
