# Supplementary material for: Characterization of poplar growth-regulating factors and analysis of their function in leaf size control
Source: BMC Plant Biol. 2020 Nov 5;20:509. doi: 10.1186/s12870-020-02699-4 (PMC7643314; doi:10.1186/s12870-020-02699-4)
Supplement: Supplementary file 9 — Additional file 9: Table S1. Complete gene information of PtrGRFs and AtGRFs. [file 12870_2020_2699_MOESM9_ESM.pdf]

Table S1. Complete gene information of *PtrGRFs* and *AtGRFs*.

Gene name	Locus	Gene names used by Cao *et al*. (2016)	Genomic position	Gene length	introns	CDS	Length (aa)	Mol. Wt. (kDa)	pI	POSRT predictions*
PtrGRF1/2a	Potri.007G007100	PtGRF11	Chr07: 491974..495612(-)	3639	3	1845	614	66.97	7.70	nucl: 14
PtrGRF1/2b	Potri.014G007200	PtGRF14	Chr14: 754921..758506(-)	3586	3	1824	607	66.13	6.61	nucl: 14
PtrGRF1/2c	Potri.002G115100	PtGRF05	Chr02: 8682667..8685517(+)	2851	3	1404	467	51.41	8.43	nucl: 14
PtrGRF1/2d	Potri.014G012800	PtGRF15	Chr14: 1330790..1334712(+)	3923	3	1830	609	65.99	8.39	nucl: 14
PtrGRF3/4	Potri.006G115200	PtGRF09	Chr06: 9021274..9023346(-)	2073	3	1134	377	42.00	7.63	nucl: 12, cyto: 2
PtrGRF5a	Potri.003G065000	PtGRF06	Chr03: 9379592..9381211(-)	1620	3	1023	340	37.81	7.48	nucl: 11, chlo: 2
PtrGRF5b	Potri.001G169100	PtGRF04	Chr01: 14239709..14241047(+	1339	3	1041	346	38.92	7.51	nucl: 12, plas: 1
PtrGRF6a	Potri.006G143200	PtGRF10	Chr06: 12094546..12096864(+	2319	3	1158	385	42.99	8.83	nucl: 12, plas: 1
PtrGRF6b	Potri.018G065400	PtGRF18	Chr18: 8275012..8277441(-)	2430	3	1149	382	43.35	8.43	nucl: 12, plas: 1
PtrGRF7a	Potri.012G022600	PtGRF12	Chr12: 1993050..1995730(-)	2681	3	1743	580	63.30	8.26	nucl: 14
PtrGRF7b	Potri.015G006200	PtGRF17	Chr15: 394635..396581(-)	1947	3	1581	526	57.90	8.12	nucl: 13
PtrGRF8	Potri.001G082700	PtGRF01	Chr01: 6526765..6529150(+)	2386	3	1542	513	55.71	7.61	nucl: 9, chlo: 1, cyto: 1, mito: 1, plas: 1
PtrGRF9	Potri.014G071800	PtGRF16	Chr14: 5800476..5803444(+)	2969	2	1377	458	50.35	8.90	nucl: 13
PtrGRF10a	Potri.001G132600	PtGRF03	Chr01: 10652440..10656748(-)	4309	3	1029	342	38.23	8.68	nucl: 11, chlo: 2
PtrGRF10b	Potri.003G100800	PtGRF07	Chr03: 12658360..12662332(+	3973	2	1044	347	38.47	9.07	nucl: 12, chlo: 2
PtrGRF11a	Potri.013G077500	PtGRF13	Chr13: 6675583..6681131(+)	5549	3	1023	340	37.47	7.80	nucl: 12, extr: 1
PtrGRF11b	Potri.019G042300	PtGRF19	Chr19: 4922919..4926904(-)	3986	3	987	328	36.26	8.66	nucl: 10, extr: 2, chlo: 1
PtrGRF12a	Potri.001G114000	PtGRF02	Chr01: 9120535..9121920(-)	1386	2	609	202	22.45	10.08	nucl: 8, chlo: 3, mito: 3
PtrGRF12b	Potri.003G118100	PtGRF08	Chr03: 14134312..14135447(-)	1136	2	606	201	22.31	9.92	nucl: 11, plas: 1.5, golg_plas: 1.5
AtGRF1	AT2G22840		Chr2:9728756..9731301(+)	2546	3	1593	530	56.40	9.68	nucl: 14
AtGRF2	AT4G37740		Chr4:17725337..17727730(-)	2394	3	1608	535	58.58	8.89	nucl: 14
AtGRF3	AT2G36400		Chr2:15270088..15272823(-)	2736	3	1197	398	43.70	8.50	nucl: 13
AtGRF4	AT3G52910		Chr3:19616021..19618449(-)	2429	3	1143	380	42.53	7.37	nucl: 13
AtGRF5	AT3G13960		Chr3:4608383..4610399(+)	2017	3	1194	397	44.70	8.20	nucl: 13
AtGRF6	AT2G06200		Chr2:2426271..2427323(+)	1053	2	735	244	28.21	8.80	nucl: 13
AtGRF7	AT5G53660		Chr5:21794536..21796046(+)	1511	2	1098	365	40.41	8.18	nucl: 14
AtGRF8	AT4G24150		Chr4:12535972..12539387(+)	3416	5	1482	493	54.61	6.93	nucl: 14
AtGRF9	AT2G45480		Chr2:18745340..18747533(+)	2194	3	1290	429	48.61	8.18	nucl: 13
