# Supplementary material for: Characterization of poplar growth-regulating factors and analysis of their function in leaf size control
Source: BMC Plant Biol. 2020 Nov 5;20:509. doi: 10.1186/s12870-020-02699-4 (PMC7643314; doi:10.1186/s12870-020-02699-4)
Supplement: Supplementary file 10 — Additional file 10: Table S2. The cleavage site of PtrGRFs by miR396 in the degradome data from Tang et al. (2016). [file 12870_2020_2699_MOESM10_ESM.pdf]

Table S2. The cleavage site of *PtrGRFs* by *miR396* in the degradome data from Tang et al. (2016).

#SmallRNA	Target	Alignment Range	Cleavage Site
ptc-miR396a	PtrGRF1/2a	1103-1124	1114
ptc-miR396b	PtrGRF1/2a	1103-1124	1114
ptc-miR396c	PtrGRF1/2a	1103-1124	1114
ptc-miR396d	PtrGRF1/2a	1103-1124	1114
ptc-miR396e-5p	PtrGRF1/2a	1103-1124	1114
ptc-miR396f	PtrGRF1/2a	1103-1124	1114
ptc-miR396g-5p	PtrGRF1/2a	1103-1124	1114
ptc-miR396a	PtrGRF1/2b	1076-1097	1087
ptc-miR396b	PtrGRF1/2b	1076-1097	1087
ptc-miR396c	PtrGRF1/2b	1076-1097	1087
ptc-miR396d	PtrGRF1/2b	1076-1097	1087
ptc-miR396e-5p	PtrGRF1/2b	1076-1097	1087
ptc-miR396f	PtrGRF1/2b	1076-1097	1087
ptc-miR396g-5p	PtrGRF1/2b	1076-1097	1087
ptc-miR396a	PtrGRF1/2c	759-780	770
ptc-miR396b	PtrGRF1/2c	759-780	770
ptc-miR396c	PtrGRF1/2c	759-780	770
ptc-miR396d	PtrGRF1/2c	759-780	770
ptc-miR396e-5p	PtrGRF1/2c	759-780	770
ptc-miR396f	PtrGRF1/2c	759-780	770
ptc-miR396g-5p	PtrGRF1/2c	759-780	770
ptc-miR396a	PtrGRF1/2d	1034-1055	1045
ptc-miR396b	PtrGRF1/2d	1034-1055	1045
ptc-miR396c	PtrGRF1/2d	1034-1055	1045
ptc-miR396d	PtrGRF1/2d	1034-1055	1045
ptc-miR396e-5p	PtrGRF1/2d	1034-1055	1045
ptc-miR396f	PtrGRF1/2d	1034-1055	1045
ptc-miR396g-5p	PtrGRF1/2d	1034-1055	1045
ptc-miR396a	PtrGRF6a	663-684	674
ptc-miR396b	PtrGRF6a	663-684	674
ptc-miR396c	PtrGRF6a	663-684	674
ptc-miR396d	PtrGRF6a	663-684	674
ptc-miR396e-5p	PtrGRF6a	663-684	674
ptc-miR396f	PtrGRF6a	663-684	674
ptc-miR396g-5p	PtrGRF6a	663-684	674
ptc-miR396a	PtrGRF7b.1	387-408	398
ptc-miR396b	PtrGRF7b.1	387-408	398
ptc-miR396c	PtrGRF7b.1	387-408	398
ptc-miR396d	PtrGRF7b.1	387-408	398
ptc-miR396e-5p	PtrGRF7b.1	387-408	398
ptc-miR396f	PtrGRF7b.1	387-408	398
ptc-miR396a	PtrGRF7b.2	739-760	750
ptc-miR396b	PtrGRF7b.2	739-760	750
ptc-miR396c	PtrGRF7b.2	739-760	750
ptc-miR396d	PtrGRF7b.2	739-760	750
ptc-miR396e-5p	PtrGRF7b.2	739-760	750
ptc-miR396f	PtrGRF7b.2	739-760	750
ptc-miR396a	PtrGRF7b.3	392-413	403
ptc-miR396b	PtrGRF7b.3	392-413	403
ptc-miR396c	PtrGRF7b.3	392-413	403
ptc-miR396d	PtrGRF7b.3	392-413	403
ptc-miR396e-5p	PtrGRF7b.3	392-413	403
ptc-miR396f	PtrGRF7b.3	392-413	403
ptc-miR396a	PtrGRF7b.4	398-419	409
ptc-miR396b	PtrGRF7b.4	398-419	409

ptc-miR396c	PtrGRF7b.4	398-419	409
ptc-miR396d	PtrGRF7b.4	398-419	409
ptc-miR396e-5p	PtrGRF7b.4	398-419	409
ptc-miR396f	PtrGRF7b.4	398-419	409
ptc-miR396a	PtrGRF7b.5	533-554	544
ptc-miR396b	PtrGRF7b.5	533-554	544
ptc-miR396c	PtrGRF7b.5	533-554	544
ptc-miR396d	PtrGRF7b.5	533-554	544
ptc-miR396e-5p	PtrGRF7b.5	533-554	544
ptc-miR396f	PtrGRF7b.5	533-554	544
ptc-miR396a	PtrGRF8	806-827	817
ptc-miR396b	PtrGRF8	806-827	817
ptc-miR396c	PtrGRF8	806-827	817
ptc-miR396d	PtrGRF8	806-827	817
ptc-miR396e-5p	PtrGRF8	806-827	817
ptc-miR396f	PtrGRF8	806-827	817
ptc-miR396g-5p	PtrGRF8	806-827	817
ptc-miR396a	PtrGRF9	576-597	587
ptc-miR396b	PtrGRF9	576-597	587
ptc-miR396c	PtrGRF9	576-597	587
ptc-miR396d	PtrGRF9	576-597	587
ptc-miR396e-5p	PtrGRF9	576-597	587
ptc-miR396g-5p	PtrGRF9	576-597	587
ptc-miR396a	PtrGRF10a	570-591	581
ptc-miR396b	PtrGRF10a	570-591	581
ptc-miR396c	PtrGRF10a	570-591	581
ptc-miR396d	PtrGRF10a	570-591	581
ptc-miR396e-5p	PtrGRF10a	570-591	581
ptc-miR396f	PtrGRF10a	570-591	581
ptc-miR396g-5p	PtrGRF10a	570-591	581
ptc-miR396a	PtrGRF10b.1	400-421	411
ptc-miR396b	PtrGRF10b.1	400-421	411
ptc-miR396c	PtrGRF10b.1	400-421	411
ptc-miR396d	PtrGRF10b.1	400-421	411
ptc-miR396e-5p	PtrGRF10b.1	400-421	411
ptc-miR396f	PtrGRF10b.1	400-421	411
ptc-miR396g-5p	PtrGRF10b.1	400-421	411
ptc-miR396a	PtrGRF10b.2	494-515	505
ptc-miR396b	PtrGRF10b.2	494-515	505
ptc-miR396c	PtrGRF10b.2	494-515	505
ptc-miR396d	PtrGRF10b.2	494-515	505
ptc-miR396e-5p	PtrGRF10b.2	494-515	505
ptc-miR396f	PtrGRF10b.2	494-515	505
ptc-miR396g-5p	PtrGRF10b.2	494-515	505
ptc-miR396a	PtrGRF10b.3	506-527	517
ptc-miR396b	PtrGRF10b.3	506-527	517
ptc-miR396c	PtrGRF10b.3	506-527	517
ptc-miR396d	PtrGRF10b.3	506-527	517
ptc-miR396e-5p	PtrGRF10b.3	506-527	517
ptc-miR396f	PtrGRF10b.3	506-527	517
ptc-miR396g-5p	PtrGRF10b.3	506-527	517
ptc-miR396b	PtrGRF10b.4	400-421	411
ptc-miR396c	PtrGRF10b.4	400-421	411
ptc-miR396d	PtrGRF10b.4	400-421	411
ptc-miR396a	PtrGRF10b.4	400-421	411
ptc-miR396e-5p	PtrGRF10b.4	400-421	411
ptc-miR396f	PtrGRF10b.4	400-421	411
ptc-miR396g-5p	PtrGRF10b.4	400-421	411

ptc-miR396a	PtrGRF11a	1041-1062	1052
ptc-miR396b	PtrGRF11a	1041-1062	1052
ptc-miR396f	PtrGRF11a	1041-1062	1052
ptc-miR396g-5p	PtrGRF11a	1041-1062	1052
ptc-miR396a	PtrGRF11b	384-405	395
ptc-miR396b	PtrGRF11b	384-405	395
ptc-miR396c	PtrGRF11b	384-405	395
ptc-miR396d	PtrGRF11b	384-405	395
ptc-miR396e-5p	PtrGRF11b	384-405	395
ptc-miR396f	PtrGRF11b	384-405	395
ptc-miR396a	PtrGRF12a.1	670-691	681
ptc-miR396b	PtrGRF12a.1	670-691	681
ptc-miR396c	PtrGRF12a.1	670-691	681
ptc-miR396d	PtrGRF12a.1	670-691	681
ptc-miR396e-5p	PtrGRF12a.1	670-691	681
ptc-miR396a	PtrGRF12a.2	773-794	784
ptc-miR396b	PtrGRF12a.2	773-794	784
ptc-miR396c	PtrGRF12a.2	773-794	784
ptc-miR396d	PtrGRF12a.2	773-794	784
ptc-miR396e-5p	PtrGRF12a.2	773-794	784
ptc-miR396a	Potri.006G191800.1	1137-1157	1148
ptc-miR396b	Potri.006G191800.1	1137-1157	1148
ptc-miR396a	Potri.006G191800.2	1137-1157	1148
ptc-miR396b	Potri.006G191800.2	1137-1157	1148
ptc-miR396e-3p	Potri.008G039400.1	2861-2881	2872
ptc-miR396e-3p	Potri.008G039400.2	2869-2889	2880
ptc-miR396g-3p	Potri.010G183500.1	1723-1743	1734
ptc-miR396g-3p	Potri.010G183500.2	1895-1915	1906
ptc-miR396c	Potri.014G077100.1	863-883	874
ptc-miR396d	Potri.014G077100.1	863-883	874
ptc-miR396e-5p	Potri.014G077100.1	863-883	874
ptc-miR396c	Potri.014G077100.2	863-883	874
ptc-miR396d	Potri.014G077100.2	863-883	874
ptc-miR396e-5p	Potri.014G077100.2	863-883	874
ptc-miR396c	Potri.016G112500.1	20-39	30
ptc-miR396d	Potri.016G112500.1	20-39	30
ptc-miR396e-5p	Potri.016G112500.1	20-39	30
ptc-miR396g-3p	Potri.019G064400.1	726-746	737
ptc-miR396g-3p	Potri.019G064800.1	924-944	935
ptc-miR396e-3p	ptc-MIR396a	9_28_	19
ptc-miR396e-3p	ptc-MIR396d	18-37	28
ptc-miR396a	ptr-miR7049.1	383-403	394
ptc-miR396b	ptr-miR7049.1	383-403	394
ptc-miR396e-3p	ptr-miR7049.1	297-316	307
ptc-miR396a	ptr-miR7049.2	392-412	403
ptc-miR396b	ptr-miR7049.2	392-412	403
