# Supplementary material for: Characterization of poplar growth-regulating factors and analysis of their function in leaf size control
Source: BMC Plant Biol. 2020 Nov 5;20:509. doi: 10.1186/s12870-020-02699-4 (PMC7643314; doi:10.1186/s12870-020-02699-4)
Supplement: Supplementary file 11 — Additional file 11: Table S3. Primers used in gene cloning and qRT-PCR analysis. [file 12870_2020_2699_MOESM11_ESM.pdf]

Table S3. Primers used in gene cloning and qRT-PCR analysis.

Primer Name	Sequences (5'-3')
GRF6brtF	AGGTTTAAATTGGGACTTG
GRF6brtR	TCAATGGAATAGTATCTCTTAC
GRF7artF	CGAACTGTAAGGATGAAACC
GRF7artR	GAATCAGCCACGCCTAAA
GRF12artF	GGCTAATAAGAACTGCTCAA
GRF12artR	CTGTGTCCTTGAAATCTTT
GRF12brtF	AGTCCAAGACCAGCAATCAGC
GRF12brtR	CCACCAAGGGAAGTAGCAACAC
CYCLIN B1;1artF	AACTTTATCCCACACACCAA
CYCLIN B1;1artR	CCAACGCTTCACTTCCAA
CYCLIN B1;1brtF	AAACCTCAGCCAAGTCGCC
CYCLIN B1;1brtR	TTCAACCCCAGCCAAATCAT
EXPA11artF	GAGTCAGATTCACCATTA
EXPA11artR	TTGAGATAAGCATTAGATTG
EXPA11brtF	GGAACTATGGGAGGGGCTTGT
EXPA11brtR	CACCATCTGGAATCCGTTTGA
ACTINrtF	AAACTGTAATGGTCCTCCCTCCG
ACTINrtR	GCATCATCACAATCACTCTCCGA
UBQrtF	CGTGGAGGAATGCAGATTTT
UBQrtR	GATCTTGGCCTTCACGTTGT
GRF1/2c-F	ATGAGCAACTCATCAGTCACAGTG
GRF1/2c-R	TTAAGCATCATTTGGGGAATGAT
GRF6b-F	ATGATAATGAGTGGAGGAAACAG
GRF6b-R	TCAAGCATCATTATTAGTTCTTGAA
GRF7a-F	ATGATGACAACAGATGATGGCTT
GRF7a-R	TTAGCCTTGATTTAACCAAAGCA
GRF9-F	ATGGAGAAAAGAGTATCTGAAGAAT
GRF9-R	CTAAGAAGAAAGGTTTTTGTTTTG
GRF10b-F	ATGAATAGTGGTGGTGCAGGAG
GRF10b-R	TTAGTTATCTCGTGGAGAACGACAA
GRF11b-F	ATGAATAGTGGTGGTGCAGCAG
GRF11b-R	TCAGTTTTCTTGTGGAGAACGAGA
GRF12a-F	ATGAAATCTCAAGCCCCACC
GRF12a-R	CTAGGCGCTAGCACTACCGA
GRF12b-F	ATGGAGTCTCAAGCCCCACC
GRF12b-R	CTAGGCGCTAGCACCACTGATT
